# Learning from the microbes: exploiting the microbiome to enforce T cell immunotherapy

**DOI:** 10.3389/fimmu.2023.1269015

**Published:** 2023-09-18

**Authors:** Sarah Staudt, Kai Ziegler-Martin, Alexander Visekruna, John Slingerland, Roni Shouval, Michael Hudecek, Marcel van den Brink, Maik Luu

**Affiliations:** ^1^ Lehrstuhl für Zelluläre Immuntherapie, Medizinische Klinik und Poliklinik II, Universitätsklinikum Würzburg, Würzburg, Germany; ^2^ Institute for Medical Microbiology and Hygiene, Philipps-University Marburg, Marburg, Germany; ^3^ Department of Immunology, Sloan Kettering Institute, New York, NY, United States; ^4^ Department of Medicine, Adult Bone Marrow Transplantation Service, Memorial Sloan Kettering Cancer Center, New York, NY, United States; ^5^ Department of Medicine, Weill Cornell Medical College, New York, NY, United States

**Keywords:** microbiome, immunotherapy, immunology, cancer immune cell therapy, CAR T cell

## Abstract

The opportunities genetic engineering has created in the field of adoptive cellular therapy for cancer are accelerating the development of novel treatment strategies using chimeric antigen receptor (CAR) and T cell receptor (TCR) T cells. The great success in the context of hematologic malignancies has made especially CAR T cell therapy a promising approach capable of achieving long-lasting remission. However, the causalities involved in mediating resistance to treatment or relapse are still barely investigated. Research on T cell exhaustion and dysfunction has drawn attention to host-derived factors that define both the immune and tumor microenvironment (TME) crucially influencing efficacy and toxicity of cellular immunotherapy. The microbiome, as one of the most complex host factors, has become a central topic of investigations due to its ability to impact on health and disease. Recent findings support the hypothesis that commensal bacteria and particularly microbiota-derived metabolites educate and modulate host immunity and TME, thereby contributing to the response to cancer immunotherapy. Hence, the composition of microbial strains as well as their soluble messengers are considered to have predictive value regarding CAR T cell efficacy and toxicity. The diversity of mechanisms underlying both beneficial and detrimental effects of microbiota comprise various epigenetic, metabolic and signaling-related pathways that have the potential to be exploited for the improvement of CAR T cell function. In this review, we will discuss the recent findings in the field of microbiome-cancer interaction, especially with respect to new trajectories that commensal factors can offer to advance cellular immunotherapy.

## Introduction

The intestinal microbiome belongs to one of the most complex communities of bacterial and fungal strains and has been shown to influence both host physiology and pathophysiology, as well as malignant and non-malignant disease development. The mutualistic interaction plays an essential role in educating immune cells and tolerogenic reactions. While the first studies in the field have demonstrated that intestinal microbial colonization is a key factor for the maintenance of gut homeostasis, work on dysbiosis showed that disease development beyond the gut, such as autoimmunity in the central nervous system and allergic reactions in the lung, is also dependent on commensal factors (1–3). The crucial impact of the microbiome in cancer was highlighted first by clinical data from patients receiving bone marrow transplantations (BMTs) who developed Graft-versus-Host-Disease (GVHD) in association with a loss of diversity of the intestinal microbiome (4). Moreover, changes in the patient microbiome composition and the production of microbial metabolites have been associated with different clinical responses in regard to immune checkpoint inhibition (ICI) and chimeric antigen receptors (CAR) T cell therapy (2, 5–9). Analyses of commensal influences on tumor cells have shown the potential to promote carcinogenesis and identified intratumoral colonization as an additional layer of the tumor microenvironment (TME) (10–12). These findings highlight the microbiome as a source for novel and therapeutically relevant strategies such as rational shaping of the commensal community in patients as preconditioning before or synergistically acting intervention combined with immunotherapies (e.g. ICI or adoptive T cell transfers).

CARs and transgenic T cell receptors (TCRs) are a result of the advances in synthetic biology and genetic engineering which have led to new strategies to redirect T cell specificity towards tumor-associated antigens (TAAs) (13, 14). The adoptive cell therapy (ACT) of engineered T cells has evolved into a therapeutic approach that has become a breakthrough in cancer immunotherapy (15), capable of demonstrating substantial response rates and efficacy in advanced malignancies (16–19).

TCRs composed of an α- and β-chain that form a heterodimer embedded in the CD3 signalling complex recognize their antigen in the context of a major histocompatibility complex (MHC) on specialized antigen-presenting cells (APCs) via MHC class II or virus infected and malignant cells via MHC class I (15, 20–22). Early approaches have isolated endogenous tumor-specific T cells from tumor lesions, blood or lymph nodes of patients which were expanded *in vitro* before adoptive transfer of mono- or oligoclonal repertoires into HLA-complementary recipients with encouraging results (23–26). The establishment of gene transfers to obtain T cells with transgenic TCR in combination with gene editing technologies has made ACT more accessible for the treatment of different disease entities (27, 28) However, the unique repertoire of MHC molecules among humans restricts the broad use of conventional TCRs for therapeutic applications to certain haplotypes (29). Furthermore, the identification of appropriate target and neoantigens is a laborious process supported by novel screening technologies (30).

In contrast, CARs are designed synthetically and consist in their basic embodiment of an extracellular binding domain that is derived from an antibody’s single chain fragment variable (scFv). The antigen-specific scFvs are fused to the intracellular signaling domains of the TCR, such as the CD3ζ chain, followed by one or more co-stimulatory domains (e.g. CD28 or 4-1BB) (31–33). Instead of scFvs, also extracellular portions of ligands or receptors for the target antigen can serve as CAR binding domain (34, 35). Consequently, activation of CARs upon antigen recognition occurs in a MHC-independent manner and does not cause mismatches with endogenous TCR chains (36). Modern gene transfer methods have enabled the generation of autologous and allogeneic CAR T cells from blood for a wide range of patients (19, 37, 38).

Although both TCR and CAR T cell approaches have shown impressive clinical outcomes, there is a necessity to investigate the hurdles and mechanisms that interfere with long-term fitness and response of engineered immune cells to broaden the range of *hard-to-treat* cancers. Host-specific factors such as the tumor microenvironment (TME) are crucially affecting the response towards immunotherapies.

In this review, we discuss the capacity of the intestinal microbiome to shape the TME, the host immune state, and the molecular mechanisms of microbiome-derived metabolites that modulate T cell responses to improve the efficacy of ACTs.

## The microbiome educates host immunity

### The role of different mouse models in advancing the field of microbiota

Over the last decades, insights into host-microbiome interaction have been facilitated by the establishment of gnotobiotic animals with defined colonization as model systems. Especially the dissection of the immune system in germ-free (GF) mice has highlighted the microbiome as a crucial factor for the education of T and B cells as well as for the generation of lymphoid organs. As compared to the standard wildtype strains kept under specific pathogen-free (SPF) conditions, GF mice show reduced antibody levels and repertoire diversity, underdeveloped lymphoid structures and impaired induction of T cell memory response (39–43).

The importance of microbiome-mediated imprinting of the host immune environment was also demonstrated by approaches that aimed for the generation of mouse models that closely resemble the natural mammalian metaorganism including coevolved commensals and pathogens. Laboratory mice are a pillar of biomedical research and many discoveries in immunology (44). It has become evident that the variable reproducibility of results originates from dissonant microbiota among laboratory animal facilities, which might therefore limit the capacity of these models to predict the complexity of the human immune environment. In order to mirror the physiology of free-living mammals in contrast to the sanitized environment in classic lab animals, Rosshart and colleagues developed strains by introducing C57BL/6 embryos into wild mice, such called “wildlings” (43). These wildlings have not only been shown to stably host the natural microbiota over multiple generations and antibiotic, under both dietary and microbial challenges, but also to reflect the clinical observations that could not be predicted in the according pre-clinical studies previously.

While administration of a CD28-superagonist caused an inflammatory cytokine response instead of the originally observed Treg expansion in wildlings, administration of anti-TNF-α treatment to wildlings during lethal endotoxemia did not lead to rescue as observed in conventional animals (45–47). Efforts to characterize wildlings and minimal microbiota consortia are critical for our understanding of how the microbiome is imprinting the host immune environment as a premise for immunotherapeutic interventions (43, 48).

### The impact of microbiota in shaping the immune response in health and disease

Commensals contribute to gut homeostasis, a fine and dynamic balance of inflammatory and immunosuppressive mechanisms, by inducing the differentiation of regulatory T cells (Tregs) as gatekeepers of peripheral and mucosal tolerance. Simultaneously, members such as *segmented filamentous bacteria* (SFB) are required for the development of Th17 cells in the mucosa which both orchestrate the intestinal immune response against bacteria but were also identified as a prerequisite for the development of T cell-mediated autoimmunity. While GF mice show resistance to induction of experimental autoimmune encephalomyelitis (EAE), monocolonization with SFB is sufficient to generate mucosal Th17 cells as pathogenic drivers of central nervous system inflammation (49). Interestingly, soluble commensal metabolites have been shown to repress the SFB-mediated onset of EAE either by promoting the differentiation of Tregs or by inducing interleukin (IL)-10 as an anti-inflammatory regulator of the microenvironment (50, 51).

**Figure 1:**
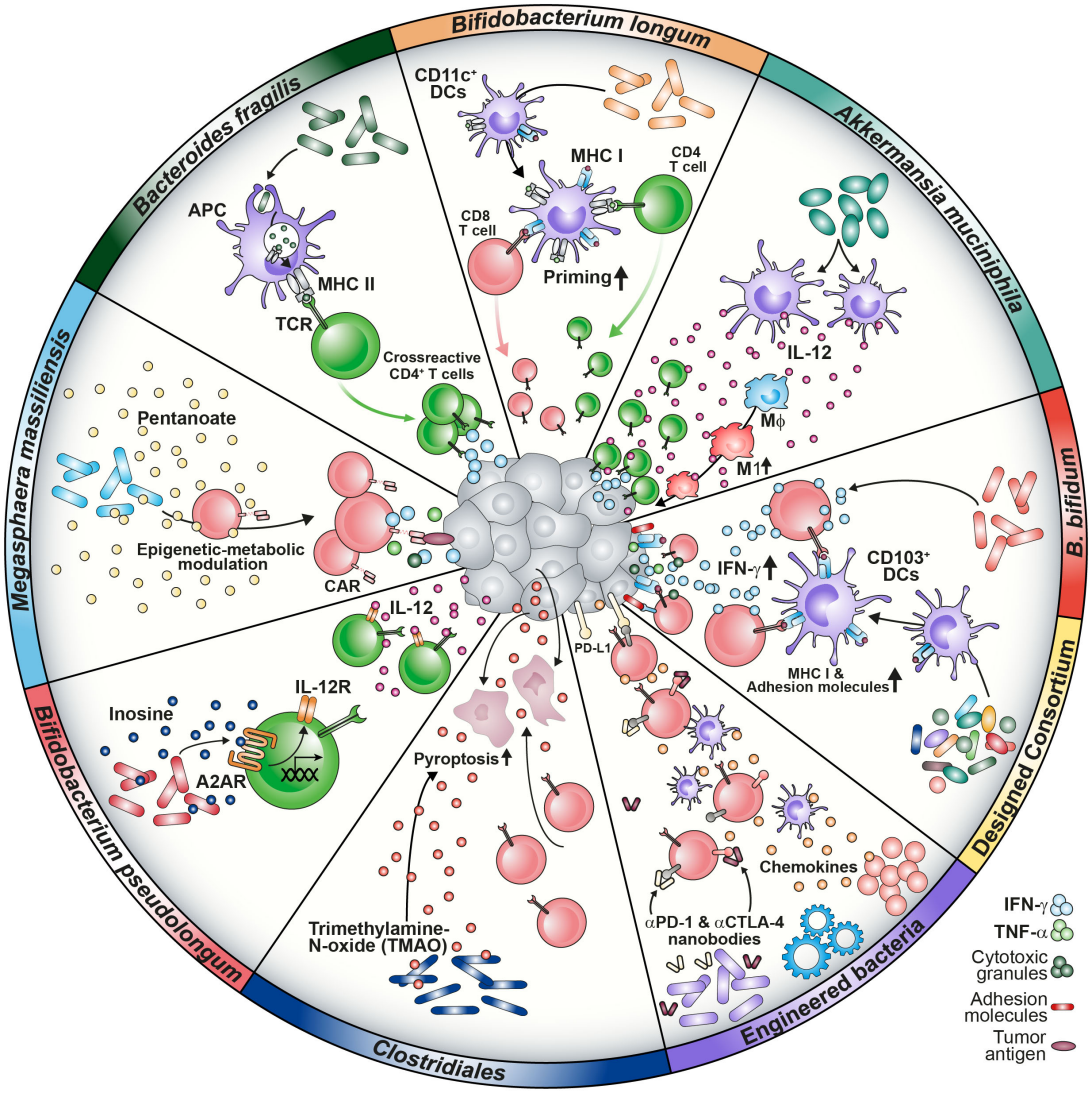
Graphical summary of microbiome-medicated mechanisms improving cancer immunotherapy approaches. Microbial composition shapes a responsive TME in several ways. Strains such as *B. longum, B. fragilis, B bifidum* and designed consortia have been found to improve T cell priming via increase of MHC class I and II molecules on DCs, tumor infiltration and IFN-γ secretion. Prevalence of *A. muciniphila* was associated with enhanced IL-12 secretion by DCs causing macrophage maturation towards the M1 phenotype. Microbial metabolites are capable of modulating T cells directly by epigenetic-metabolic reprogramming (*M. massiliensis*-derived pentanoate) or by inducing the IL-12 receptor on CD4 T cells via the inosine-A2AR axis (*B. pseudolongum*). TMAO triggers pyroptosis in tumor cells and increases CD8 T cell-mediated antitumor immunity (*Clostridiales*). Further, engineering of bacteria to produce ICI nanobodies or chemokines were reported to reprogram the TME favorably.

Also in other inflammatory diseases such as Graft-versus-Host-Disease (GVHD), expansion of certain microbiota strains was correlated with disease incidence (4). The lactose-dependent expansion of *Enterococcus* after allogeneic hematopoietic cell transplantation (allo-HCT) in the intestine of gnotobiotic animals enhanced GVHD severity which was attenuated by dietary depletion of the disaccharide. Similarly, allo-HCT patients with low capabilities to absorb lactose were dominantly colonized with *Enterococcus* post-antibiotically (4). Despite being involved in shaping an inflammatory environment, the microbiome is a crucial provider of nutrients promoting hematopoietic recovery after bone marrow transplantation (52). Commensal depletion reduced both dietary energy uptake and visceral fat storage and led to worse lymphocyte and neutrophil recovery as compared to WT hosts. The phenotype was restored by the administration of sucrose, compensating the caloric deficits. Interestingly, work on calorie restriction has reported the enrichment of *Bifidobacterium bifidum* which resulted in increased antitumor immunity by IFN-γ^+^ CD8 T cells infiltrating the TME (**Figure 1**) (53). These investigations have contributed to the hypothesis that an interplay between several commensal factors is involved in maintaining physiological homeostasis further strengthening the thought of dysbiosis as a detrimental cause of pathophysiology.

### Microbiome-mediated TME modulation can favor immunotherapy outcome

Preclinical studies have contributed to our knowledge about the microbiome-cancer axis and the outcome of immunotherapy. Several aspects, such as the priming and activation of immune cells or the attraction to the tumor site, have been described (54). The investigation of correlations between bacterial strains and immunotherapy outcome was driven by the initial observation that the different microbial composition of laboratory mice derived from either TAC of JAX resulted in different tumor growth kinetics and had an impact on ICI response. Sivan and colleagues identified *Bifidobacterium longum* as commensal that enhanced T cell priming by dendritic cells (DCs) and their accumulation in tumors, which improved tumor treatment. Administration of the strain further acted synergistically with α-PD-L1 therapy (55). Moreover, *Bacteroides fragilis* was associated with an improved response to anti-cytotoxic T-lymphocyte-associated protein-4 (α-CTLA-4) blockade due to the development of *B. fragilis*-specific T cells combating the tumor (**Figure 1**) (6). This implies that antigen mimicry can be mechanistically involved in inducing antitumor immunity (56).

Analysis of clinical data correlated antibiotics treatment with a decreased survival of NSCLC patients and indicated a positive link between *Akkermansia muciniphila* abundance and response to α-PD-1 ICI. Of note, fecal microbiota transplantation (FMT) from α-PD-1 responders into either GF or microbiome-depleted animals reflected the clinical outcome in preclinical mouse models in an IL-12-dependent manner (5) (**Figure 1**). In accordance, first FMT studies have promoted the ICI response in immunotherapy-refractory melanoma patients (57).

The importance of IL-12 as a TME-modulating factor was recently demonstrated by CAR T cells engineered to release the pro-inflammatory cytokine upon CAR engagement (58, 59). Recruitment and enhancement of macrophage function were accompanied by improved killing of tumor cells with antigen-loss in a TNF-α-dependent manner (59). Integration of IL-12 into the CAR exodomain conferred NK-like killing properties to CD8 T cells (58). Of note, intratumoral delivery of the cytokine both supported the antitumor activity of CAR T cells and the repolarization of the TME by attracting infiltrating CD4 T cells, further highlighting that microbiome-induced IL-12 could boost engineered T cell function (60).

Similarly, the synergism between CAR T cells and α-PD-1 blockade was shown in HER2- and GD2-dependent preclinical models (61, 62). Engineering strategies mediating T cell-intrinsic PD-1 pathway interference were tested to improve the potency of CAR T cells (63, 64). However, although the first results from clinical trials combining ACT and ICI have obtained encouraging results as well, no cases with significant increase in CAR T expansion and persistence were reported upon checkpoint blockade (65–67). Based on these insights, future studies might include the microbiome modulation to enhance the response towards ICI in combination with CAR T cell therapy.

The promising results from ICI studies have encouraged research teams to design bacterial consortia capable of shaping the TME favorably to improve T cell response. In accordance with that concept, Tanoue and colleagues generated a defined consortium of 11 strains derived from the human microbiome capable of increasing the frequency of IFN-γ^+^ CD8 T cells. Colonization with the selected strains improved resistance against *Listeria monocytogenes* infection as well as ICI in a CD103^+^ DC-dependent manner (68) (**Figure 1**). Besides the importance of IFN-γ for T cell differentiation towards anti-viral and –tumoral responses, this cytokine has been shown to act as a crucial modulator of the TME (69–71). Kantari-Mimoun and colleagues described an IFN-γ-dependent two-step process that involves the ICAM-1/LFA-A1 axis, allowing CAR T cells to traffic from the periphery into the tumor islets (72). Similarly, Larsson and colleagues showed the importance of the IFN-γ pathway, highlighting that enhancing CAR T cell binding affinity and adhesion to target cells might increase solid tumor responses (73).

**Figure 2:**
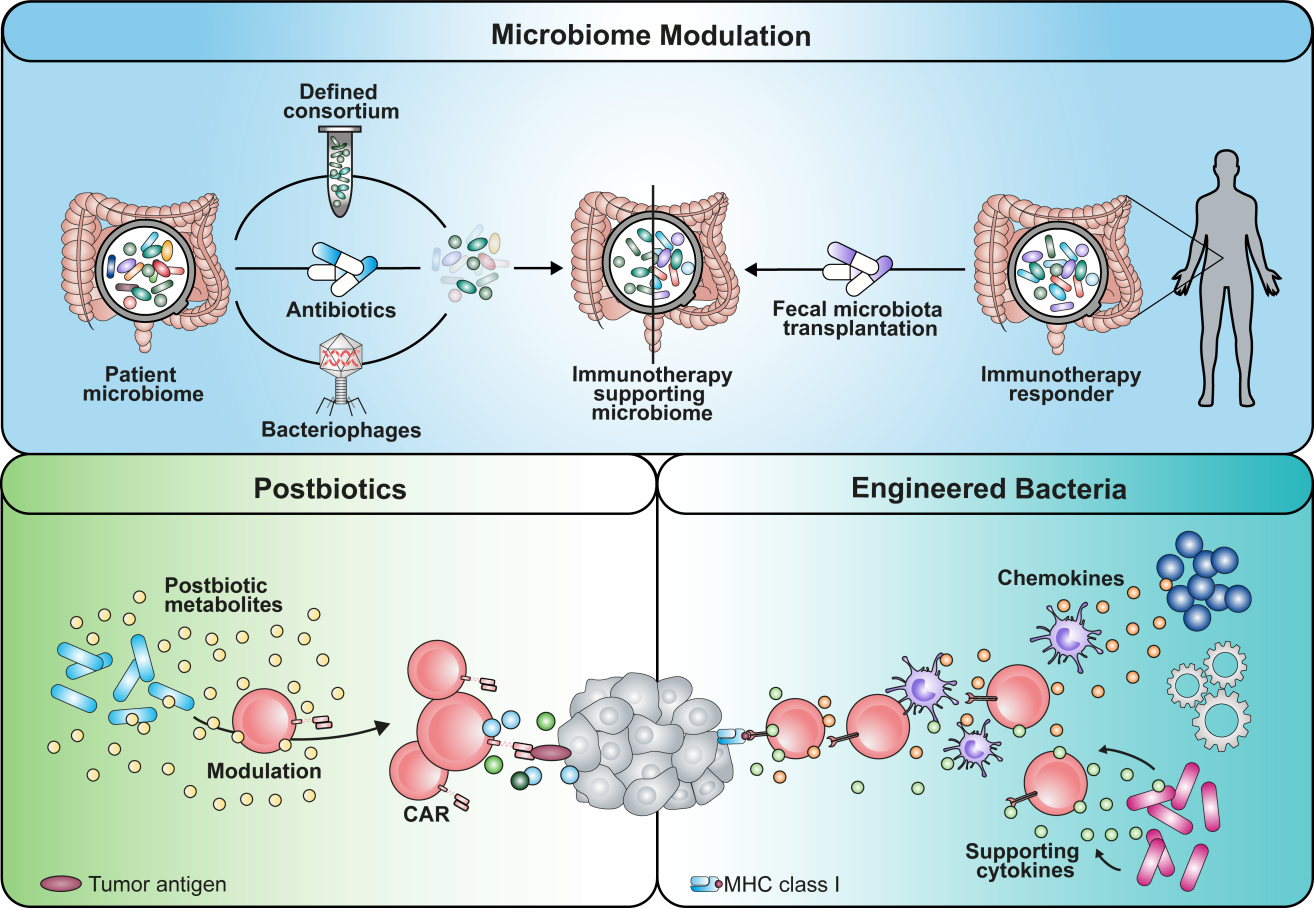
Graphical summary of potential implementation strategies that can be used to apply microbiome-derived mechanisms in cancer immunotherapy. An immunotherapy-favoring microbiome modulation could be achieved by either depleting strains using antibiotics or phages with certain selectivity. Alternatively, establishment of beneficial commensals could be enabled by transplantation of a defined consortium or fecal microbiota of responding patients. Similarly, engineered bacteria reprogramming the TME with soluble mediators is a potential avenue. Additionally, the use of microbial metabolites as postbiotic drugs has the potential to boost immunotherapy.

Hence, the design of microbial consortia could be considered as a strategy to prime the TME for efficient CAR T infiltration and response in tumor islets (**Figures 1**, **2**).

## Intratumoral microbiome can be utilized to improve T cell-mediated antitumor immunity

A study investigating the human cancer microbiome uncovered that intratumoral bacteria are present in various solid malignancies, such as breast and ovarian cancer, lung and pancreatic tumor tissues. Surprisingly, these bacteria could be detected even in those that have no direct communication with the external environment (e.g., glioblastoma or bone tumors) (74). This discovery gave rise to the term *oncobiome* (74, 75). The presence of tumor-associated bacteria in immunosuppressive micro niches points to a highly organized colonization of transformed tissues that affects the behavior of tumor and immune cells (12). Intriguingly, it was postulated that the cell-associated members of the intratumoral microbiota could drive the migration of cancer cells and impact on the cellular heterogeneity of the TME. Intestinal bacteria and some oral bacteria have been found in colorectal carcinoma (CRC) samples. The orally found commensal *Fusobacterium nucleatum* was shown to translocate to the colon. Enrichment of the bacterium in the tumor tissue has led to worse radiotherapy outcome and promoted colorectal carcinogenesis (10, 76, 77). Intratumoral colonization was shown to directly modulate the efficacy of chemotherapeutic agents due to metabolization of pharmacologic compounds, highlighting the microbiome as a TME factor affecting several layers of tumor-host interaction (78). Recently, Bender and fellows showed that the as probiotic considered strain *Lactobacillus reuteri* can translocate to the tumor tissue in a preclinical melanoma model where it is driving the effector function of CTLs depending on a tryptophan-enriched diet (79). Additionally, colonization of the cancerous mammary gland with *Clostridiales* genera was associated with an activated immune microenvironment due to the presence of bacterial-derived trimethylamine N-oxide (TMAO) that improved immunotherapy response (80) (**Figure 1**). These results underline not only the involvement of nutrition and diet in shaping the oncobiome, but also a potential for intratumoral bacteria to modulate TME favorably for immune cells (79, 81).

In support of this concept, the engineering of microbes capable of secreting α-PD-L1 or α-CTLA-4 nanobodies to mediate ICI or chemokines such as CXCL16 and CCL20 to recruit CTLs and DCs to the malignancy site has been shown to elicit antitumor effects (82, 83) (**Figures 1**, **2**). Two recently published studies focusing on CD19 CAR T cell intervention for B cell malignancy patients found that antibiotic treatment prior to CAR T cell infusion was correlated with adverse outcomes (8, 9). Especially the administration of piperacillin/tazobactam, imipenem/cilastatin and meropenem (P-I-M) within a 4 week window pre-CAR T cell treatment was associated with worse survival and increased neurotoxicity (9). About 80% of patients receiving CD19 CAR T cell therapy experience cytokine release syndrome (CRS) or immune effector cell-associated neurotoxicity syndrome (ICANS). While the analysis of a prospective cohort revealed an association between *Clostridia* species and day 100 complete response, the results highlighted that both CAR T efficacy and CRS/ICANS are influenced by the microbiome composition which might be responsible for the immune cell activation state and environment. As *Clostridia* are well known to produce metabolites such as short-chain fatty acids (SCFAs), the lack of bacterial factors in antibiotics-treated patients might contribute to the described adverse outcomes (50, 84). Further, Stein-Thoeringer and colleagues correlated exposure to wide-spectrum antibiotics with decreased survival and CAR T responsiveness with *Bacteroides, Ruminococcus, Eubacterium* and *Akkermansia* (8).

An immunostimulating effect of the commensals, which is dampened by antibiotic treatment, could also be attributed to other factors like Toll-like receptor (TLR) ligands. Work from Paulos et al. has demonstrated that adoptive transfer of tumor-specific CD8 T cells into lymphodepleted host show reduced antitumor activity upon reduction of the microbiota via antibiotics or neutralization of serum LPS (85). Similarly, irradiated TLR4-deficient mice showed worse tumor repression compared to WT controls. Notably, the administration of ultrapure LPS to lymphodepleted hosts enhanced the potency of ACT in a syngeneic model. In line with these findings, we have investigated an association between the abundance of the bacterial groups and relapse/progression of disease after allogeneic hematopoitic-cell transplantation (allo-HCT). Analysis of stool samples from 541 patients admitted for allo-HCT was performed via 16S sequencing during a two year-follow up after the treatment. Interestingly, the analysis revealed an association between abundance of *Eubacterium limosum* and less relapse/disease progression post allo-HCT (86).

A study performed by Hu and colleagues explored the role of the microbiome on CAR T cell-mediated CRS in relapsed/refractory multiple myeloma (87). By collecting microbiota samples before CAR-T infusion, during infusion prior to cytokine storm development, during active cytokine storm, and up to fourteen days after CAR-T treatment, the group could link severe CRS to the reduction of *Bifidobacteria* abundance and observed a decline in microbiota diversity after CAR T cell administration. Interestingly, the decline was accompanied by higher abundance of *Enterococcus* and *Actinomyces*. Complete response in patients was associated with enrichment of *Bifidobacterium* and *Prevotella* species. The study is another example of how commensal composition is connected to ACT outcome.

Based on these data, the understanding of TME-modifying probiotic bacteria can contribute to new combinatorial cancer therapies using engineered microbes or defined consortia in synergy with adoptively transferred T cells that benefit from improved function and response.

## Adoptive cell therapy can be tuned with commensal effector molecules

The microbiome harbors an enormous repertoire of genes encoding for enzymatic pathways which enable the production of small molecules that can serve as postbiotics (**Figure 2**). These commensal metabolites serve as second messengers that, in contrast to most gut-resident bacteria, can cross the epithelium and diffuse through the lamina propria reaching the systemic circulation (88). Thus, soluble microbial metabolites can modulate immune and non-immune cells in both proximity and distance to the gut bridging the gap between host and microbiome. Initially, the mechanistic investigation of short-chain fatty acids (SCFAs), a dominant class of commensal products, as physiological inducers of Tregs in the intestine has drawn attention to their therapeutic potential.

We have demonstrated that the treatment of CD8 T cells with the SCFA pentanoate enhances the expression of CTL-associated genes such as IFN-γ and TNF-α via epigenetic-metabolic reprogramming. Pentanoate acts as a specific inhibitor of histone deacetylase (HDAC) class I and impacts the mTOR pathway as one of the key metabolic regulators (84, 89). In *in vivo* experiments, pentanoate-treated TCR-transgenic T cells showed increased antitumor activity and persistence in syngeneic solid tumor models. These features were conferred to CAR T cells which elicited superior tumor control in contrast to the untreated control group in a pancreatic TME. Moreover, acetate, which is abundantly produced by commensals and tumor cells, is capable of fueling the T cell metabolism in glucose-restricted CD8 T cells. The SCFA enhances the IFN-γ secretion in an acetyl-CoA synthetase (ACSS)-dependent manner (90). Although the modulation of histones is one of the intensively studied mechanisms in the SCFA field, their impact on DNA methylation has remained rather understudied. An effect of butyrate has been described as an inductor of ERK phosporylation which in turn down-regulates the DNA methyltransferase DNMT1 (91). Consequently, demethylation of tumor suppressor genes was observed. However, more in-depth research in immune cells needs to be done to assess the mechanisms involved in microbiome-mediated modulation of DNA methylation which bears a strong potential for ACTs. Recent work has highlighted DNA methylation as reprogramming mechanism capable of determining CAR T cell fate (92).

Remarkably, although tumor-derived lactate has been identified as a glycolysis-derived metabolite with immunosuppressive characteristics in the TME that induces M2-like polarization in TAMs, a new study has reported an increase in CD8 T cell stemness and antitumor response following lactate treatment (93, 94). Mechanistically, lactate mediated these effects by suppressing HDAC activity, which caused hyperacetylation at H3K27 of the Tcf7 super enhancer locus. The subsequent Tcf7 gene expression was associated with a stem-like phenotype (95). These results raise a question about the involvement of the dominant commensal-derived lactate and *Lactobacillus* strains in tuning T cell function and immune cell activation (4, 94).

Denk and colleagues identified that urolithin A (UA), a metabolite derived from the conversion of ellagitannins by the gut microbiome, is improving mitochondrial health. By engaging the Pink1-Pgam5 axis, UA triggered mitophagy and compensatory mitochondrial biogenesis causing the differentiation of T memory stem cells (TSCMs) with superior CD8 antitumor immunity (96).

Not only CD8 T cells, but also CD4 T cells appear to be modulated by microbial metabolites. The analysis of *Bifidobacterium pseudolongum* in the context of immunotherapy revealed that the production of inosine induced the expression of the IL-12 receptor on CD4 T cells via the adenosine A2A receptor (**Figure 1**). Translocation of inosine through a leaky gut barrier into the systemic circulation improved antitumor response. The crucial role of CD4 CAR T cells was recently pointed out by Melenhorst and colleagues, who observed that CD4^+^ CAR T cells dominated the CAR T cell population of patients with decade-long leukemia remission at later monitoring time points. Those cells maintained cytotoxic characteristics and proliferation (97, 98).

These insights suggest that commensal metabolites with the capacity to reprogram host immune cells can be utilized as postbiotic physiologic drugs to potentiate effector function and memory features of ACT products. Furthermore, commensal-based adjuvants might provide new tools to achieve long-term function and antitumor reactivity of CAR T cells.

## Future directions

The microbiome is a complex network of microorganisms that directly and indirectly influence host physiology and immunological homeostasis. The education of the host immune system is a prerequisite not only for a functional defense against infectious diseases and malignant transformation but also for promoting tolerance. Studies have demonstrated that response and resistance to cancer immunotherapy is dependent on the immune cell activity within the TME, which can be modulated by the intestinal or translocated microbiome. On one hand, these findings allow us to think about the microbial composition as a predictive indicator that could guide the choice of the immunotherapeutic approach to achieve long-lasting remission (9, 87, 99).

On the other hand, understanding how commensals prime the TME can be an important asset to shape the surrounding for endogenous and engineered T cells beneficially. More detailed knowledge about the affected cell populations in the TME, such as myeloid-derived suppressor cells and tumor-associated macrophages, is necessary. Based on these insights, different strategies have the potential to be implemented in clinical setting (**Figure 2**). The first approach might include the administration of defined microbial consortia as probiotics or of personalized antibiotic treatment regimens that shape the microbiome favorably (9, 68). Using strain-specific phages could be an alternative technology with higher precision compared to antibiotic treatment (100). In line with this concept, fecal microbiota transplantation (FMT) from donors that have shown response to immunotherapy might be capable of overcoming resistance mechanisms. As a second approach, engineering of microbes for intratumoral colonization can be used synergistically with ACTs (82, 83). These are able to reprogram the TME by either secreting modulatory factors themselves (e.g. antibodies, cytokines) or recruiting endogenous immune cells using chemokines (101, 102). However, we need to keep in mind that the inter-individual diversity of the microbiome remains a challenge in the process of creating the optimal formulation for a designer consortium. Further research needs to be performed to clarify whether a “one-size fits all” drug is more suitable rather than a personalized medicine approach.

While TME reprogramming is a potential avenue to obtain a less suppressive milieu, rewiring immune cells themselves to overcome the latter is an additional course. Commensal-derived metabolites that bridge the host-microbiome interaction are a powerful source of physiological molecules with drug-like properties, also considered as postbiotics (e.g. SCFAs). While their production within the host can be modulated and utilized by diet, metabolite libraries might be valuable to screen and identify novel tools to improve ACTs and immune cell engineering (7, 79, 84, 96, 103).

A crucial prerequisite to obtain the knowledge required might be the availability of preclinical models that reflect the human microbiome and its influences on host immunity as well as immunotherapy. Previous studies have pointed out that the transfer of human microbiota into gnotobiotic mice was capable to reflect the ICI responses in patients (5, 6). Also the establishment of wilding colonies had a strong predictive value with regards to the outcome of clinical studies (43). These revolutionary steps are still in need of standardization to assure reproducibility and accessibility of “humanized” gnotobiotic animals for a broad community of researchers (43, 104).

For a better understanding of the host-microbiome interaction, an interdisciplinary approach is needed that synergizes bioinformatics and systems biology, molecular microbiology, immunology and biotechnology in order to facilitate bench-to-bedside and back translation. As research labs are highly specialized and have limited capacities to cover both extensive clinical data collection from international cohorts and molecular studies of the microbiome-mediated effects, innovative ecosystems are required to move the field forward. Within the EU-funded research consortium T2EVOLVE, a public-private partnership of CAR and TCR T cell key opinion leaders, we aim to accelerate the development and improve access to engineered T cell therapy​ (105, 106). Synergies with the microbiome-CAR T consortium CARTOMICS will establish an infrastructure that boosts collaborative multidisciplinary work between partners, stakeholders, cell engineering approaches and microbiome-associated facets that guide the next-generation of cellular immunotherapy.

## Author contributions

SS: Writing – original draft, Writing – review & editing. KZ-M: Writing – original draft, Writing – review & editing. AV: Writing – original draft, Writing – review & editing, Funding acquisition. JS: Writing – original draft, Writing – review & editing. RS: Writing – original draft, Writing – review & editing. MH: Writing – original draft, Writing – review & editing. MvdB: Writing – original draft, Writing – review & editing. ML: Writing – original draft, Conceptualization, Funding acquisition, Visualization.
